# Personality and Age of Male National Team of Ukraine in Kyokushin Karate—Pilot Study

**DOI:** 10.3390/ijerph19127225

**Published:** 2022-06-13

**Authors:** Paweł Piepiora, Bogdan Kindzer, Justyna Bagińska, Wojciech J. Cynarski

**Affiliations:** 1 Faculty of Physical Education and Sports, Wroclaw University of Health and Sport Sciences, 51-612 Wrocław, Poland; 2 Faculty of Physical Education and Sport, Lviv State University of Physical Culture Named after Ivan Boberskyj, 79000 Lviv, Ukraine; bogdankindzer@ukr.net; 3 Faculty of Economics and Management, University of Business in Wrocław, 53-238 Wrocław, Poland; justyna.baginska@handlowa.eu; 4 Institute of Physical Culture Studies, University of Rzeszów, 35-959 Rzeszów, Poland; ela_cyn@wp.pl

**Keywords:** sports psychology, health psychology, Big Five, combat sports, Ukrainian sportsmen

## Abstract

This article is a continuation of the research on personality in combat sports in karate. The authors’ goal was to verify the relationship between personality and age of kyokushin karate practitioners. The male national team of Ukraine in karate kyokushin (*N* = 7) participated in the personality study with the use of the Big Five model. The NEO-FFI (NEO Five-Factor Inventory) Personality Questionnaire was applied as a research tool and the package of statistical methods IBM SPSS Statistics 27.0 (IBM Polska, Warszawa, Poland) was used to compute the research results. The study showed that there were differences in the intensity of openness to experiences between individual samples only at the level of the statistical trend. Masters showed a higher level of openness to experiences in relation to juniors (*p* = 0.081) and seniors (*p* = 0.097). Also, a negative and strong correlation between the intensity of neuroticism and conscientiousness among the respondents was noted. A conclusion was drawn that, with age, karatekas probably manifest greater openness to experience, which is the result of their sports experience, high sports level and pro-health values of karate. On the other hand, good emotional adaptation of karatekas is strictly related to conscientiousness.

## 1. Introduction

Karate continues to maintain its popularity. Its entry into the Olympic disciplines contributed to this, which indeed began to supplant the popularity of mixed martial arts [1]. Another important factor is the return to the full contact formula, through the dynamic development of contact karate organizations and the participation of outstanding masters of various styles in these fights [2]. It is worth noting that pop culture has not remained passive either and one can also notice the return of karate themes in films, computer games and lifestyle [3]. Hence, it is reasonable to continue research on personality among karate practitioners, because personality is shaped by the influence of the environment in which individuals function [4]. The influence of society is obvious [5]. In the case of karate, we can talk about the phenomenon of karate culture [6,7], which is characteristic for adepts of this martial art, combat sport and self-defense system in one [8]. Karate culture manifests itself in the everyday functioning of karatekas in the society [9]. Therefore, the relationship between karate culture and society, and the effect of this relationship in the form of formed personality of karatekas [10], is an important area of research in the field of sport and health psychology. Previous research in this area shows that the personality profiles of karatekas are similar to those of other athletes [11], but, at the same time, they are not the same for combat sports [12], individual sports [13] and team sports [14]. The personality of karatekas, regardless of the adopted training goals, is shaped by their physical activity [15]. This is noticeable in sports [16], physical education [17], tourism [18] and recreation [19]. Therefore, there is no difference in personality profiles between kata and kumite athletes [20]. Things are different, however, upon comparing personality profiles of sports champions, who are significantly distinguished by lower neuroticism in relation to the rest of athletes [21]. However, combat sports champions have significantly better emotional control than champions from other sports groups [22]. It is also important that, alongside with sport experience, the personality of athletes is more clearly defined: lower neuroticism and higher extraversion, openness to experience, agreeableness and conscientiousness [23]. Moreover, female kyokushin karate practitioners have higher neuroticism in relation to males [24]. It is worth mentioning that kyokushin karate is characterized by kata [25] and kumite competitions in the knockdown system [26]. It is considered by many karate experts as the toughest one [27,28], as the victory over the opponent can be obtained by knocking him out or by having more judges’ indications after the regular fighting time has elapsed [29].

The cited studies focused mainly on seniors (20–29 years). So far, the relationship between personality and age of champion level kyokushin karate practitioners has not been verified. Therefore, it has been assumed that it would be reasonable to examine if there are differences between personality profiles of juniors (15–19 years), seniors (20–29 years) and masters (30–39 years) in this sport discipline, since the obtained results may be important for the field of sport and health psychology. Therefore, it has been decided to verify the following hypothesis: there are differences between personality profiles and age among juniors, seniors and masters of kyokushin karate.

## 2. Methodology

### 2.1. Tested Persons

The study included 7 male representatives of Ukraine in kyokushin karate (the entire male representation). These athletes train professionally. Their biggest international success was in kumite divisions (−60 kg, −65 kg, −70 kg, −75 kg, −85 kg, −90 kg, +90 kg) during the European Karate Kyokushin Championship. Five of the discussed karatekas hold 1st dan master’s degrees, the other two hold 2nd dan master’s degrees. The study was conducted on 19 February 2021 during the national team training camp of Ukraine at Lviv State University of Physical Culture named after Ivan Boberskyj. All subjects consented to participate in the study and agreed that the research results will be used for scientific purposes. The research was supposed to be continued at the University of Rzeszow, Poland, during the next training camp of the national team of Ukraine, but due to the COVID-19 pandemic it did not take place. Unfortunately, during the process of lifting pandemic restrictions in Poland, the Russian Federation invaded Ukraine on 24 February 2022 and the examined individuals were enrolled into the Ukrainian armed forces. Thus, continuing further research work on the male national team of Ukraine in kyokushin karate is not possible. However small, this research sample (*N* = 7) is of the highest quality. It included 3 juniors (18 years old, each), 2 seniors (21–23 years old) and 2 masters (34–39 years old).

### 2.2. Research Method

In this study, a five-factor model of personality called the Big Five was applied. It includes five main personality traits: neuroticism, extraversion, openness to experience, agreeableness, and conscientiousness, which allow for their separate classification. The formal criteria for the traits of the five-factor model of personality were formulated by Costa and McCrae in the course of their discussion of the basic dimensions of personality [30,31,32,33,34]. Neuroticism has been shown to be a dimension reflecting emotional adjustment in relation to emotional imbalance, or emotionality in terms of negative emotions. Neuroticism includes six formally distinguished components. These are: anxiety (tendency to react with tension and fear, nervousness, and a tendency to worry), angry hostility (tendency to experience anger and irritability, although not necessarily expressed outwardly), depression (tendency to experience guilt, sadness, helplessness, and loneliness), impulsiveness (inability to control desires and drives), vulnerability (susceptibility to stress expressed as inability to cope with stress, tendency to react with a sense of helplessness and panic in difficult situations), self-consciousness (social anxiety or low self-esteem, embarrassment and feeling of confusion in the presence of others). Extraversion, on the other hand, is a dimension that characterizes the quality and quantity of social interactions and the level of activity, energy, and ability to experience positive emotions. Extraversion also includes six formally distinguished components: gregariousness (the extent and amount of contact maintained with other people), warmth (the ability to maintain close relationships with other people, friendliness), assertiveness (dominance and leadership tendencies), activity (pace, vigor, energy, the need to be busy and engaged), excitement-seeking (seeking excitement and stimulation), and positive emotions (the tendency to respond with positive emotions). Openness to experience, on the other hand, is a dimension that describes an individual’s tendency to seek out and positively value life experiences, tolerance of novelty, and cognitive curiosity. Openness to experience includes six components. These are: fantasy (fantasy and vivid, creative imagination), aesthetics (aesthetic sensitivity, interest in art), feelings (openness to emotional states of other people), actions (active search for new stimuli), ideas (intellectual curiosity, philosophical interests), values (readiness to analyze social, political and religious values, the opposite of dogmatism). Agreeableness is a dimension that describes a positive or negative attitude toward other people, an interpersonal orientation manifested in altruism in relation to antagonism, experienced in feelings, thoughts, and actions. Agreeableness includes as components: trust (the belief that others have honest intentions versus skepticism and cynicism and the belief that others may be dishonest and dangerous), straightforwardness (straightforwardness, honesty, and social naiveté versus the tendency to manipulate other people), altruism (the tendency to focus on other people’s needs and to help them versus egocentrism), compliance (how one responds to interpersonal conflicts: restraint of aggressiveness, meekness and gentleness, and the tendency to “forgive and forget” versus aggressiveness and competitive tendencies), modesty (realistic attitude toward oneself, no tendency to favor oneself versus belief in one’s own superiority and narcissistic tendencies) tendermindedness (displaying affection and sympathy for others, supporting charitable organizations and actions versus rationality and matter-of-factness in dealing with others, and “low sensitivity to human social and welfare problems”). Moreover, conscientiousness is a dimension that characterizes an individual’s degree of organization, persistence, and motivation in goal-oriented activities and describes a person’s attitude toward work. The components of conscientiousness are: competence (belief in one’s ability to cope in life versus belief in lack of ability and lack of skills to cope with tasks), order (orderliness, diligence, and neatness versus lack of regularity and order in life and action), dutifulness (strict adherence to one’s principles), achievement striving (high level of aspiration and strong motivation to achieve success in life, high commitment to work, but also a tendency to workaholism versus lack of ambition, self-discipline (ability to self-motivate in order to finish the tasks started, even if they are not attractive versus the tendency to abandon tasks before their completion), deliberation (tendency to carefully consider the problem before making a decision and starting to act in relation to impulsiveness when making decisions, but also spontaneity and the ability to make quick decisions if necessary) [35,36].

### 2.3. Research Tool

The NEO-FFI Personality Questionnaire [37] was used to diagnose personality traits in the Big Five model. The personality traits measured by the NEO-FFI characterize a normal personality. In this sense, the clinical interpretation of NEO-FFI is not adopted, but this tool is used for scientific research in sport psychology. The questionnaire items consist of 60 self-descriptive statements, the truthfulness of which in relation to the self is assessed by the respondent on a five-point scale. These items form five scales that measure the Big Five model. The NEO-FFI is reliable, valid, and internally consistent. It has sten norms for five age groups (15–19 years, 20–29 years, 30–39 years, 40–49 years, 50–80 years), developed for both genders. For our study, the norms for males aged 15–19 for juniors, 20–29 for seniors, and 30–39 for masters were applied [37].

### 2.4. Procedure

The study was conducted according to the guidelines of the Declaration of Helsinki. Each respondent agreed to participate in the study after being informed about the objectives and principles of the study, the expected effects and possible benefits for those taking part in the study. The respondents were also acquainted with the mode and possibility of withdrawal from participation in the study. In addition, the respondents were informed that they have the opportunity to ask questions and get answers. All respondents consented to the processing of data related to their participation in the study and the use of study results for research purposes. The study was conducted in a room isolated from noise. Respondents were given one hour to respond to statements from the NEO-FFI personality questionnaire. Upon completion of the study, the participants’ data were coded.

### 2.5. Statistical Analysis

Statistical analyses were performed using IBM SPSS Statistics 27.0 software. The Shapiro–Wilk test to check the normality of data, one-way ANOVA analyses of variance, and r Pearson correlation analyses were performed with the software. The level of statistical significance in the following section was assumed at α = 0.05.

## 3. Results

In the first step of the analyses, basic descriptive statistics were performed along with normality of distribution tests for the tested personality traits. The detailed results of the analysis are presented in Table 1. The analysis of normality of distribution using the Shapiro–Wilk test showed that the distribution of the variables is close to a normal distribution (*p* > 0.050), except for the trait of neuroticism (*p* = 0.049). However, upon analyzing the value of skewness and kurtosis, it can be concluded that the distribution of the neuroticism variable only slightly deviates from the normal distribution.

Next, it was examined whether kyokushin karate practitioners differed in the intensity of personality traits by age. The subjects were divided into three samples based on age. A junior sample (*n* = 3), a senior sample (*n* = 2), and a masters sample (*n* = 2) were identified. Before proceeding with the analyses, the samples were checked for equality using the chi-square test. The analysis revealed no statistically significant differences in the number of individuals per group, χ^2^(2) = 0.29, *p* = 0.867. In order to test for differences in individual personality traits between athletes, a series of one-way analyses of variance were performed, because the distribution of personality traits was close to a normal distribution and the age groups tested were characterized as equivalent. Within the analyses, no statistically significant differences were observed in the intensity of neuroticism, extraversion, agreeableness, and conscientiousness according to age. However, differences were observed in the intensity of openness to experience between samples, *F*(2.6) = 7.10, *p* = 0.048. However, post hoc analysis with the Bonferroni test revealed that the masters’ sample had a higher intensity of openness to experience (*M* = 31.50) than the juniors’ sample (*M* = 26.67) only at the level of statistical trend (*p* = 0.081). A similar effect (*p* = 0.097) was observed for the difference between the masters’ sample and the seniors’ sample (*M* = 26.50). Detailed results of the analyses are presented in Table 2.

In the last step of the analyses, it was examined whether there was a relationship between the different personality traits. For this purpose, Pearson’s *r* correlation analysis was performed. Within the analysis, a negative and strong (*r* > 0.6) relationship was observed between the intensity of neuroticism and conscientiousness. No significant relationships were observed between the remaining personality traits. Detailed results of the analysis are presented in Table 3.

## 4. Discussion

The obtained results of the research did not show statistically significant differences between personality profiles and age of the male national team of Ukraine in kyokushin karate. The research hypothesis was verified and it was observed that there were no differences between personality profiles and the age of juniors, seniors, and masters among kyokushin karatekas. Only in the dimension of openness to experience was a statistical trend noticed: with age the intensity of this trait increases. People with high openness to experiences are curious about the phenomena of both the external and internal world and have a richer life in terms of the number of experiences. In contrast, individuals with low openness to experience are conventional in their behavior and conservative in their views. The key assumption of the study was that, with age and gained life experience (including sports level and professional sports experience), the kyokushin karatekas’ openness to experience increases. Therefore, the trend in openness to experiences is an (admittedly) small but high-quality sample that should be treated as a reflection of sports experience of karatekas [23]. The study subjects are also high-level sportsmen with international sport success in kyokushin karate, so sport level is also important [38]. It is worth noting that openness to experience is associated with intellectual characteristics. The relationship has more to do with divergent thinking ability and creativity than with convergent thinking ability and intelligence, because many highly intelligent people are not open to experience, and, at the same time, many people who are open to experience are not particularly intellectually capable [35]. In this sense, practicing kyokushin karate as a conscious physical activity has a positive impact on health in physical and mental aspects. Physical, because through training and competition, karate students improve their physical fitness [39] and technical-tactical skills [40]. Mentally, because through karate culture [7] adepts become more resilient to difficult life situations [41,42]. Individuals with higher levels of openness to experience are more unconventional, willing to question authority, independent in judgment, and driven to explore new social and ethical ideas. Therefore, the studied karatekas may be perceived as more mature and healthier. A high or low level of openness to experience depends mainly on the influences of a given environment and situation. In our case, the tested persons are professional Ukrainian kyokushin karate practitioners and, from this perspective, the positive effect of karate training on physical [43] and mental [44] health in relation to sport experience [45] and sport level [46] should be considered valid.

In addition, a relationship between neuroticism and conscientiousness among the studied persons has been noticed. The lower the level of neuroticism in male representatives of Ukrainian kyokushin karate, the higher their conscientiousness level. In general, in the personality of sports champions, on the one hand, a significant role is attributed to low neuroticism [21,47], but on the other hand, there are also data that indicate a significantly high level of extraversion [48] or a high level of conscientiousness [49]. This supports the fact that personality dimensions are cross-cultural [50,51,52]. From a health psychology perspective, illness symptoms and malaise are predominantly associated with high neuroticism, but do not necessarily imply mental or psychosomatic illness. In contrast, individuals with low neuroticism show the opposite tendency to deny health problems [35]. From this perspective, neuroticism is a predictor of individual health status, because it is associated with health attitudes and behaviors [36]. However, also conscientiousness is related to the individual’s manifestation of positive and objectively beneficial health behaviors [30]. High conscientiousness is related to the level of performance of professional tasks and satisfaction with professional achievements [35]. In this sense, the correlation of low neuroticism and high conscientiousness in the personality of Ukrainian karatekas relates to the ability to predict professional success and associations with the mental and physical health of the subjects. This is consistent with the personality profiles of karate champions [10,12,22]. It should be noted that the coach has a contribution to the formation of the personality of karatekas [53]. Psychological and pedagogical influences occur at the stage of training [54] and competing in karate [55]. This includes both the period of athletes’ full performance [56] and the period of injury healing [57]. In this sense, the relationship of low neuroticism and high conscientiousness manifests itself as a consequence of the athlete’s mental recovery from injury [58]. Therefore, the results of this study are important for sport and health psychology.

At this point, the limitations of the study should be noted. Due to the small research sample, the results cannot be applied to the entire population of Ukrainian kyokushin karatekas. They refer only to this case study. They are a foundation for further research on the personality of karatekas and, therefore, should be considered as a pilot study. In the following research, the obtained results should be verified with the largest possible population of Ukrainian karatekas, including females, and be related to athletes from other sports disciplines.

The results of this study can be used in sport psychology through the optimal choice of methods, forms and means of training during the mental preparation of karatekas for sports competition. On the other hand, in the area of health psychology, the obtained results indicate that a healthy personality can be developed as a result of practicing karate at a high level.

## 5. Conclusions

There are no statistically significant differences between the personality profiles of the male national team of Ukraine in kyokushin karate and their age group (juniors, seniors and masters). A statistical trend was noted, however, in openness to experience and a relationship between the personality traits of the subjects: the lower the neuroticism, the higher the conscientiousness. The data indicate that, with age, karatekas are more open to experiences, which goes along with their gaining of sport experience, high sport level and health-promoting values of karate. In addition, good emotional adjustment of karatekas is associated with high levels of conscientiousness.

## Figures and Tables

**Table 1:** Basic descriptive statistics for personality traits.

	*M*	*Mdn*	*SD*	*Sk.*	*Kurt.*	*Min*	*Max*	*S–W*	*p*
Neuroticism	21.29	24.00	5.82	−1.07	−0.68	12.00	27.00	0.81	0.049
Extraversion	30.43	31.00	6.16	0.10	−1.38	22.00	38.00	0.92	0.448
Openness to experience	28.00	28.00	2.71	0.42	−1.06	25.00	32.00	0.91	0.382
Agreeableness	27.57	26.00	3.87	0.41	−1.50	23.00	33.00	0.92	0.459
Conscientiousness	31.71	30.00	3.68	−0.24	−1.19	26.00	36.00	0.88	0.214

*M*—mean, *Mdn*—median, *SD*—standard deviation, *Sk.*—skewness, *Kurt.*—kurtosis, *Min*—minimum value, *Max*—maximum value, *S–W*—Shapiro–Wilk test, *p*—statistical significance of the Shapiro–Wilk test.

**Table 2:** Results of a series of one-way analyses of variance.

*Variables*	Juniors		Seniors		Masters		*F*	*p*
	*M*	*SD*	*M*	*SD*	*M*	*SD*		
Neuroticism	24.00	1.00	20.50	9.19	18.00	8.48	0.57	0.607
Extraversion	34.00	6.93	26.50	6.36	29.00	4.24	0.95	0.460
Openness to experience	26.67	1.53	26.50	2.12	31.50	0.71	7.10	0.048 ^ab^
Agreeableness	25.00	1.73	30.50	2.12	28.50	6.36	1.52	0.323
Conscientiousness	31.67	2.89	30.50	6.36	31.71	3.68	0.17	0.852

*M*—mean, *SD*—standard deviation, *F*—ANOVA test results, *p*—significance, ^a^—Bonferroni post hoc test difference between the junior sample and the senior sample at *p* = 0.081, ^b^—Bonferroni post hoc test difference between the senior sample and the masters sample at *p* = 0.097.

**Table 3:** Correlations between the intensity of individual personality traits.

		Neuroticism	Extraversion	Openness to Experience	Agreeableness
Extraversion	Pearson’s *r*	−0.18			
	significance	0.706			
Openness to experience	Pearson’s *r*	−0.61	−0.12		
	significance	0.143	0.798		
Agreeableness	Pearson’s *r*	−0.47	−0.11	0.06	
	significance	0.290	0.814	0.892	
Conscientiousness	Pearson’s *r*	−0.827	0.18	0.60	−0.01
	significance	0.022	0.707	0.153	0.983

## Data Availability

The authors confirm that the data supporting the findings of this study are available within the article.

## References

[1] Kalina R.M., Barczyński B.J. Long way to the Czestochowa Declarations 2015: HMA against MMA. Proceedings of the 1st World Congress on Health and Martial Arts in Interdisciplinary Approach.

[2] Cynarski W.J. (2014). The European karate today: The opinion of experts. Ido Mov. Cult. J. Martial Arts Anthropol..

[3] Kusnierz C., Cynarski W.J., Gorner K. (2017). Social reception and understanding of combat sports and martial arts by both school students and adults. Ido Mov. Cult. J. Martial Arts Anthropol..

[4] Witkowski K., Piepiora P., Leśnik M., Migasiewicz J. (2017). Social status of karate and personal benefits declared by adults practicing karate. Arch. Budo Sci. Martial Arts Extrem. Sports.

[5] Aronson E., Aronson J. (2020). The Social Animal.

[6] Piepiora P., Piepiora Z. The karate culture as the regulator of interdependence between permitted level of violence in different kumite systems and personality of contestants. Proceedings of the Human and Social Sciences at the Common Conference (HASSACC).

[7] Piepiora P., Szmajke A., Migasiewicz J., Witkowski K. (2016). The karate culture and aggressiveness in kumite competitors. Ido Mov. Cult. J. Martial Arts Anthropol..

[8] Piepiora P. (2021). Kompendium Karate.

[9] Piepiora P., Petecka A. (2020). Personality profile of women practising contact sports using the example of karate kyokushin competitors and handball players. Ido Mov. Cult. J. Martial Arts Anthropol..

[10] Piepiora P., Witkowski K., Piepiora Z. (2018). Personality profiles of karate masters practising different kumite styles. Arch. Budo.

[11] Piepiora P., Witkowski K. (2018). Personality traits of competitive athletes according to type of pressure exerted on opponents. S. Afr. J. Res. Sport Phys. Educ. Recreat..

[12] Piepiora P., Witkowski K. (2020). Personality profile of combat sports champions against neo-gladiators. Arch. Budo.

[13] Piepiora P. (2021). Personality profile of individual sports champions. Brain Behav..

[14] Piepiora P. (2021). Assessment of Personality Traits Influencing the Performance of Men in Team Sports in Terms of the Big Five. Front. Psychol..

[15] Piepiora P., Kozak M., Witkowski K. (2020). Personality profile of athletes who declare that they train kyokushin karate as a martial art. Arch. Budo Sci. Martial Arts Extrem. Sports.

[16] Pacesova P. (2021). Cognitive and Executive Functions of Young Men regarding Sport Activity and Personality Traits. Sustainability.

[17] Newsome A., Kilpatrick M., Mastrofini G., Wilson K.E. (2021). Personality Traits and Physical Activity: Helping Exercise Professionals Maximize Client Outcomes. ACSM’s Health Fit. J..

[18] Corneliu S.C., Constantin R.B., Bagińska J. (2012). Study regarding the evolution of the body’s adaptation to the specific effort of hiking in physical education and sports students, during the practical courses in touristic activities. Gymnasium.

[19] Sterkowicz S., Franchini E. (2009). Testing motor fitness in karate. Arch. Budo.

[20] Piepiora P., Petre L., Witkowski K. (2021). Personality of karate competitors due to their sport specialization. Arch. Budo.

[21] Piepiora P., Piepiora Z. (2021). Personality determinants of success in men’s sports in the light of the Big Five. Int. J. Environ. Res. Public Health.

[22] Piepiora P., Witkowski K. (2020). Self-defence as a utilitarian factor in combat sports, modifying the personality of athletes at a champion level. Arch. Budo Sci. Martial Arts Extrem. Sports.

[23] Piepiora P., Piepiora Z., Bagińska J. (2022). Personality and Sport Experience of 20–29-Year-Old Polish Male Professional Athletes. Front. Psychol..

[24] Piepiora P., Komarnicka N., Gumienna R., Maśliński J. (2022). Personality and gender of people training in Kyokushin karate and kickboxing. Ido Mov. Cult. J. Martial Arts Anthropol..

[25] Kindzer B., Saienko S., Diachenko A. (2018). Ability of kata “Sanchin” Kyokushinkai karate to quickly restore the bodies of karate sportsmen after significant physical activity. J. Phys. Educ. Sport.

[26] Piepiora P., Migasiewicz J., Witkowski K. (2016). The traditional karate training and sports fight systems of kumite. Rocz. Nauk. Wyższej Szkoły Wych. Fiz. I Tur. W Białymstoku.

[27] Oyama M. (1970). Advanced Karate.

[28] Oyama M. (1978). Essential Karate.

[29] Arneil S. (1980). Kumite Rules for Karate Matches Using Knockdown Scoring System.

[30] McCrae R.R., Costa P.T. (1987). Validation of the five-factor model of personality across instruments and observers. J. Personal. Soc. Psychol..

[31] Eysenck H.J. (1991). Dimensions of personality: 16, 5 or 3?—Criteria for a taxonomic paradigm. Personal. Individ. Differ..

[32] Costa P.T., McCrae R.R. (1992). Four ways Five Factors are basic. Personal. Individ. Differ..

[33] Eysenck H.J. (1992). Four ways Five Factors are not basic. Personal. Individ. Differ..

[34] Costa P.T., McCrae R.R. (1992). Reply to Eysenck. Personal. Individ. Differ..

[35] McCrae R.R., Costa P.T. (1997). Personality trait structure as a human universal. Am. Psychol..

[36] Costa P.T., Terracciano A., McCrae R.R. (2001). Gender differences in personality traits across cultures: Robust and surprising findings. J. Personal. Soc. Psychol..

[37] Costa P.T., McCrae R.R. (2007). NEO-FFI Personality Inventory.

[38] Piepiora P., Kwiatkowski D., Bagińska J., Agouridas D. (2021). Sports Level and the Personality of American Football Players in Poland. Int. J. Environ. Res. Public Health.

[39] Navickaitė A., Thomas G. (2022). Strength and Conditioning Considerations for Kyokushin Karate Athletes. Strength Cond. J..

[40] Szczęsna A., Błaszczyszyn M., Pawlyta M. (2021). Optical motion capture dataset of selected techniques in beginner and advanced Kyokushin karate athletes. Sci. Data.

[41] Sterkowicz S., Blecharz J., Sterkowicz-Przybycień K. (2012). Stress in sport situations experienced by people who practice karate. Arch. Budo.

[42] Kostorz K., Sas-Nowosielski K. (2021). Aggression Dimensions Among Athletes Practising Martial Arts and Combat Sports. Front. Psychol..

[43] Akbaş A., Brachman A., Gzik B., Bacik B. (2021). The objective assessment of striking force in combat sports using sport-specific measurement devices—A review. Arch. Budo.

[44] Vveinhardt J., Kaspare M. (2022). The Relationship between Mindfulness Practices and the Psychological State and Performance of Kyokushin Karate Athletes. Int. J. Environ. Res. Public Health.

[45] Błaszczyszyn M., Szczęsna A., Pawlyta M., Marszałek M., Karczmit D. (2019). Kinematic Analysis of Mae-Geri Kicks in Beginner and Advanced Kyokushin Karate Athletes. Int. J. Environ. Res. Public Health.

[46] Niewczas M., Piepiora P., Cynarski W.J. (2021). Attitudes of training youths towards karate on the example of the Polish national team in the youth category. Arrancada.

[47] Steca P., Baretta D., Greco A., D’Addario M., Monzani D. (2018). Associations between personality, sports participation and athletic success. A comparison of Big Five in sporting and non-sporting adults. Personal. Individ. Differ..

[48] Allen M.S., Mison E.A., Robson D.A., Laborde S. (2021). Extraversion in sport: A scoping review. Int. Rev. Sport Exerc. Psychol..

[49] Mirzaei A., Nikbakhsh R., Sharififar F. (2013). The relationship between personality traits and sport performance. Eur. J. Exp. Biol..

[50] Allen M.S., Greenlees I., Jones M. (2011). An investigation of the five-factor model of personality and coping behaviour in sport. J. Sports Sci..

[51] Allen M.S., Greenlees I., Jones M. (2013). Personality in sport: A comprehensive review. Int. Rev. Sport Exerc. Psychol..

[52] Allen M.S., Laborde S. (2014). The role of personality in sport and physical activity. Curr. Dir. Psychol. Sci..

[53] Witkowski K., Proskura P., Piepiora P. (2016). The role of the combat sport trainer in the education of youth—A reference to the traditional standards and perception of understanding of the sport in the life of the individual and socjety. Arch. Budo Sci. Martial Arts Extrem. Sports.

[54] Cynarski W.J., Słopecki J., Dziadek B., Boschen P., Piepiora P. (2021). Indicators of targeted physical fitness in judo and jujutsu—Preliminary results of research. Int. J. Environ. Res. Public Health.

[55] Zarzycki A., Witkowski K., Pożarowszczyk B., Kumorek M., Kisilewicz A., Smoter M., Korpal Ł., Piepiora P., Kawczyński A. (2017). Changes in muscle stiffness as the effect of karate tournament fight. Arch. Budo Sci. Martial Arts Extrem. Sports.

[56] Vovkanych L., Kindzer B., Fedkiv M. (2022). Perspectives for improvement of karate stance performance on the basis of electromyogram analysis. Ido Mov. Cult. J. Martial Arts Anthropol..

[57] Bolach B., Witkowski K., Piepiora P., Sokólski R., Bolach E. (2016). Injuries and overloads in combat sports exemplified by Thai boxing and judo. J. Combat. Sports Martial Arts.

[58] Cynarski W.J., Niewczas M. (2017). Perception and attitude towards Karate among the members of the Polish Junior representation squad: Diagnostic survey. Arrancada.

